# How to reduce household costs for people with tuberculosis: a longitudinal costing survey in Nepal

**DOI:** 10.1093/heapol/czaa156

**Published:** 2020-12-20

**Authors:** Suman Chandra Gurung, Bhola Rai, Kritika Dixit, Eve Worrall, Puskar Raj Paudel, Raghu Dhital, Manoj Kumar Sah, Ram Narayan Pandit, Tara Prasad Aryal, Govinda Majhi, Tom Wingfield, Bertie Squire, Knut Lönnroth, Jens W Levy, Kerri Viney, Job van Rest, Andrew Ramsay, Rafaely Marcia Santos da Costa, Buddha Basnyat, Anil Thapa, Gokul Mishra, Julia Moreira Pescarini, Maxine Caws, Noemia Teixeira de Siqueira-Filha

**Affiliations:** Birat Nepal Medical Trust: Lazimpat, Ward No. 2, Kathmandu, Nepal; Department of Clinical Sciences, Liverpool School of Tropical Medicine, Pembroke Place, Liverpool L3 5QA, UK; Birat Nepal Medical Trust: Lazimpat, Ward No. 2, Kathmandu, Nepal; Birat Nepal Medical Trust: Lazimpat, Ward No. 2, Kathmandu, Nepal; Karolinska Institutet, Department of Global Public Health, 171 77, Stockholm, Sweden; Department of Clinical Sciences, Liverpool School of Tropical Medicine, Pembroke Place, Liverpool L3 5QA, UK; Birat Nepal Medical Trust: Lazimpat, Ward No. 2, Kathmandu, Nepal; KNCV Tuberculosis Foundation, Maanweg 174, 2516 AB, Den Haag, the The Netherlands; Birat Nepal Medical Trust: Lazimpat, Ward No. 2, Kathmandu, Nepal; Birat Nepal Medical Trust: Lazimpat, Ward No. 2, Kathmandu, Nepal; Birat Nepal Medical Trust: Lazimpat, Ward No. 2, Kathmandu, Nepal; Birat Nepal Medical Trust: Lazimpat, Ward No. 2, Kathmandu, Nepal; Birat Nepal Medical Trust: Lazimpat, Ward No. 2, Kathmandu, Nepal; Department of Clinical Sciences, Liverpool School of Tropical Medicine, Pembroke Place, Liverpool L3 5QA, UK; Karolinska Institutet, Department of Global Public Health, 171 77, Stockholm, Sweden; Department of Clinical Sciences, Liverpool School of Tropical Medicine, Pembroke Place, Liverpool L3 5QA, UK; Karolinska Institutet, Department of Global Public Health, 171 77, Stockholm, Sweden; KNCV Tuberculosis Foundation, Maanweg 174, 2516 AB, Den Haag, the The Netherlands; Karolinska Institutet, Department of Global Public Health, 171 77, Stockholm, Sweden; Australian National University, Research School of Population Health, College of Health & Medicine, Canberra, ACT 2600, Australia; KNCV Tuberculosis Foundation, Maanweg 174, 2516 AB, Den Haag, the The Netherlands; University of St Andrews, College Gate St Andrews, KY16 9AJ, UK; Oswaldo Cruz Foundation, Centro de Pesquisa Aggeu Magalhaes, Av. Professor Moraes Rego, s/n – Cidade Universitária – Recife/PE, CEP 50.740-465, Brazil; Oxford University Clinical Research Unit, P. O. Box 26500, Kathmandu, Nepal; National Tuberculosis Control Centre, Thimi, Bhaktapur, Nepal; Birat Nepal Medical Trust: Lazimpat, Ward No. 2, Kathmandu, Nepal; Department of Clinical Sciences, Liverpool School of Tropical Medicine, Pembroke Place, Liverpool L3 5QA, UK; Oswaldo Cruz Foundation, Centro de Integracao de Dados e Conhecimentos para Saude, Rua Mundo, 121, Trobogy, Salvador - Bahia, CEP 41745-715, Brazil; Birat Nepal Medical Trust: Lazimpat, Ward No. 2, Kathmandu, Nepal; Department of Clinical Sciences, Liverpool School of Tropical Medicine, Pembroke Place, Liverpool L3 5QA, UK; Department of Clinical Sciences, Liverpool School of Tropical Medicine, Pembroke Place, Liverpool L3 5QA, UK; University of York, Department of Helarh Sciences, Heslington, YO10 5DD, York, UK

**Keywords:** Tuberculosis, case finding, costs, catastrophic costs, Nepal

## Abstract

The aim of this study was to compare costs and socio-economic impact of tuberculosis (TB) for patients diagnosed through active (ACF) and passive case finding (PCF) in Nepal. A longitudinal costing survey was conducted in four districts of Nepal from April 2018 to October 2019. Costs were collected using the WHO TB Patient Costs Survey at three time points: intensive phase of treatment, continuation phase of treatment and at treatment completion. Direct and indirect costs and socio-economic impact (poverty headcount, employment status and coping strategies) were evaluated throughout the treatment. Prevalence of catastrophic costs was estimated using the WHO threshold. Logistic regression and generalized estimating equation were used to evaluate risk of incurring high costs, catastrophic costs and socio-economic impact of TB over time. A total of 111 ACF and 110 PCF patients were included. ACF patients were more likely to have no education (75% vs 57%, *P* = 0.006) and informal employment (42% vs 24%, *P* = 0.005) Compared with the PCF group, ACF patients incurred lower costs during the pretreatment period (mean total cost: US$55 vs US$87, *P* < 0.001) and during the pretreatment plus treatment periods (mean total direct costs: US$72 vs US$101, *P* < 0.001). Socio-economic impact was severe for both groups throughout the whole treatment, with 32% of households incurring catastrophic costs. Catastrophic costs were associated with ‘no education’ status [odds ratio = 2.53(95% confidence interval = 1.16–5.50)]. There is a severe and sustained socio-economic impact of TB on affected households in Nepal. The community-based ACF approach mitigated costs and reached the most vulnerable patients. Alongside ACF, social protection policies must be extended to achieve the zero catastrophic costs milestone of the End TB strategy.

**KEY MESSAGES**

- The community-based active case finding (ACF) strategy reduced tuberculosis (TB) pretreatment cost for patients by 65% (ACF: US$20; passive case finding: US$58).
- The ACF strategy reached the most vulnerable and disadvantaged population and can contribute to promoting equity of access to TB services.
- The longitudinal costing survey evidenced an enduring and severe socio-economic impact in both groups. The unemployment rate increased by 72% during the intensive phase of treatment, and patients continually reported food insecurity throughout treatment.

## Introduction

Tuberculosis (TB) kills more people each year than any other single infectious disease and principally affects the most vulnerable populations in low- and middle-income countries (World Health Organization, 2019). The socio-economic consequences of TB are often severe, and many TB-affected households are pushed into extreme poverty due to the high out-of-pocket expenditures and income lost during the search for TB diagnosis and treatment. Structural causes commonly found in developing countries, such as seasonal economy, poor access to healthcare facilities and low education, can also contribute to worsening the economic hardship faced by TB-affected households (Whitehead and Dahlgren, 2007; Barrett and Carter, 2013)*.* The World Health Organization’s (WHO) End TB strategy (World Health Organization, 2015b) has established ambitious goals to advance towards TB elimination, including zero catastrophic costs for TB affected households, to be achieved by 2020. Catastrophic TB costs are defined by WHO as total costs of TB diagnosis and care above 20% of the household’s annual income (World Health Organization, 2015a). The latest Global TB report published by the WHO in 2010 shows that the zero catastrophic costs milestone will not be achieved by the end of 2020. National costing surveys conducted in 12 high burden countries have shown that the percentage of TB-affected families facing catastrophic costs ranged from 27% in Kenya to 83% in Timor-Leste for all forms of TB. As catastrophic cost is an important indicator to estimate the economic burden of TB and evaluate access to healthcare, the WHO has established a monitoring framework including this indicator as essential to monitor beyond 2020. The organization has also recommended universal health coverage to improve access to high-quality TB diagnosis and treatment and social protection schemes as priority policies to achieve the zero catastrophic costs milestone (World Health Organization, 2020).

Another recommendation to monitor the progress towards the zero catastrophic costs milestone is the implementation of patient cost surveys (World Health Organization, 2019). Several countries have now conducted national or local surveys by adopting cross-sectional (Nhung *et al.*, 2018) or longitudinal approaches (Foster *et al.*, 2015); addressing costs of TB and co-morbidities such as HIV/AIDS (Mudzengi *et al.*, 2017; de Siqueira Filha *et al.*, 2018) and diabetes (Arnold *et al.*, 2016); and comparing active case finding (ACF) vs passive case finding (PCF) (Morishita *et al.*, 2016; Gurung *et al.*, 2019; Muniyandi *et al.*, 2020). Modelling studies have also been developed to determine the impact of specific TB interventions on patient costs (Verguet *et al.*, 2017). However, evidence regarding the impact of community-based ACF on patient costs using the more detailed longitudinal approach is still lacking.

Nepal is one of the poorest countries in Asia with 15% of the population classified as extremely poor (World Bank, 2018). The 2018–19 national TB prevalence survey in Nepal showed an incidence rate of 245/100 000, which is much higher than previous estimates (Government of Nepal *et al.*, 2020). This means that less than half (46%) of the incident TB cases in Nepal in 2019 (69 000) were diagnosed or notified via the government system, with approximately 40 000 ‘missing’ cases occurring annually (Government of Nepal *et al.*, 2020). Strategies to reach these missing TB cases are urgently needed. ACF (World Health Organization, 2013) reaching out into communities to actively screen and diagnose people with TB is one strategy to reduce this case notification gap, decrease morbidity and mortality and interrupt community transmission.

The IMPACT TB was launched in 2017 to implement a community-based ACF model in four districts of Nepal and to increase the evidence for optimal ACF scale-up policies. Here, we report the results of the cost analysis that compared costs and socio-economic impact of TB in patients diagnosed through ACF with the standard National TB Programme (NTP) PCF.

## Methods

### Study design and setting

A longitudinal TB Patient Cost Survey was conducted between April 2018 and October 2019 in four districts of Nepal covering both rural and urban areas, hilly and lowland Terai regions: Dhanusha (population 754 777; 2.8% of the national population) and Mahottari (population 627 580; 2.4% of the national population) in Province 2, and Makwanpur (population 420 477; 1.6% of the national population) and Chitwan (population 579 984; 2.2% of the national population) in Province 3. Makwanpur, Mahottari and Chitwan districts are considered high burden TB districts, i.e. case notification rate (CNR) >120, and Dhanusha is classified as medium TB burden district, i.e. CNR between 75 and 120. These districts reported 2061 TB cases in 2018, which accounts for 11% of all reported TB cases in Nepal (Ministry of Health & Population *et al.*, 2018).

### Sample size calculation and sampling

At the time of study design, there were no studies comparing ACF and PCF incurred patient costs on which to base an effect size estimate. Therefore, we took a pragmatic approach and sample size was based upon previous TB patient costing surveys in other countries that showed a sample of 100 patients is sufficient to capture the spectrum of TB patient costs incurred (Ukwaja *et al.*, 2012). Allowing for an expected attrition rate of 20% in the study sites, 121 patients were therefore recruited for each study arm (ACF and PCF). TB patients diagnosed through ACF and PCF were registered at IMPACT TB database and at the treatment registers at Directly Observed Treatment Short-course (DOTS) centres, respectively. A research associate checked the list of patients diagnosed in both databases monthly and consecutively selected participants until reaching the target sample size in each arm. Patients were recruited from April 2018 to January 2019.

### Inclusion criteria

Adults, ≥18 years old, with laboratory bacteriological confirmed pulmonary TB (new, retreatment or relapse), resident of Nepal, with written informed consent provided were eligible for this study. Drug-resistant TB patients were excluded from this study due to time and budget constraints.

### Interventions

PCF is the current practice implemented by the NTP in Nepal. Symptomatic individuals seek healthcare by self-presentation at healthcare facilities, which includes a network of health posts, primary health centres and government district hospitals. PCF pathway includes (1) patients are aware of their symptoms and access health facilities, (2) patients are evaluated by health workers who recognize the symptoms of TB and (3) patients are referred to diagnostic centres to collect sputum sample and perform a TB test (World Health Organization, 2013). In Nepal, sputum smear microscopy is the standard diagnostic test within the NTP, with GeneXepert available in some centres and currently reserved for priority groups. The NTP is prioritizing the scale-up of GeneXpert testing (Government of Nepal *et al.*, 2017).

Full details of the community-based ACF model applied are given elsewhere (Gurung *et al.*, 2019). A brief description is given below.

ACF was implemented through a community-based approach, identifying presumptive TB cases via symptom screening of social contacts of all TB index cases in the district. In addition, TB camps were implemented in remote communities. Household contacts of TB index cases were screened in these districts by a separate Global Fund supported intervention. The IMPACT TB study applied smear microscopy for TB testing in Mahottari and Makwanpur and GeneXpert MTB/RIF in Chitwan and Dhanusha.

Index TB patients were identified at the government health facilities and were contacted to collect information about their social contacts. After consent of the index patient, community health workers (CHW) scheduled a visit to the social contacts to perform symptom screening (cough, fever, night sweats or weight loss). Presumptive cases were invited to undergo TB testing.

TB camps were performed in areas with a high number of TB cases notified and remote areas with no healthcare access and informal settlements. Door-to-door symptom screening by CHW identified presumptive cases for TB testing at the camp.

All positive TB cases were enrolled on standard TB treatment at the nearest government DOTS facility.

### Data collection tool

The WHO TB Patient Costs Survey (KNCV Tuberculosis Foundation, World Health Organization and Japan Anti-Tuberculosis Association, 2008) was adapted, translated into Nepali and piloted in 16 patients prior to use in this study. The survey collected socio-economic data, direct medical costs (e.g. drugs, tests, medical fees), direct non-medical costs (e.g. transportation, accommodation and food) and indirect costs (e.g. lost time and income loss) and information on the social and economic impact of TB. After piloting, the survey was used by trained CHWs to conduct paper-based interviews at the location preferred by the patient, usually the residence of the patient or at a health facility. Interviewers followed a standard operating procedure manual developed by the project team. Completed questionnaires were then reviewed by a research associate and district program coordinators. CHWs were advised to contact patients to clarify or correct any missing or incomplete information. Participants were compensated for their time (∼60 minutes) with 500 Nepalese rupees (NPR) (∼US$4.5) for each interview.

### Patient costs, time horizon

Patient costs were collected at three time points. The first interview was conducted during the intensive phase (between 2 weeks and 2 months of treatment initiation) and collected data on costs incurred during pre-TB diagnosis (since the onset of TB symptoms) and treatment period until the date of the interview. Two subsequent interviews collected information on costs incurred during TB treatment, covering the time since the preceding interview. The second interview was applied during the continuation phase of treatment (between 3 months and 4 months) and the third at the end of treatment (sixth month of treatment). Therefore, costs incurred during TB illness from the time of symptom onset (self-reported by patients) to the time of TB treatment completion were calculated.

### Data entry and analysis

Questionnaires were entered by trained study staff into a dedicated study database designed by IMPACT TB consortium partners.

#### Socio-economic profile

The living standard was assessed using the indicators recommended by the government of Nepal to evaluate multidimensional poverty, i.e. education level, and proportion of patients included in the study with access to electricity, drinking water, sanitation and asset ownership (Government of Nepal and Oxford Poverty and Human Development Initiative, 2018).

#### Patient costs

Mean costs with 95% confidence intervals (CIs) and median costs with the interquartile range were estimated by cost type (direct medical, direct non-medical and indirect costs) and by treatment period (pretreatment and treatment). Total costs were calculated by summing all costs incurred during the pretreatment and treatment periods. Direct costs were calculated by summing all costs in each category (medical and non-medical). Indirect costs comprised lost income and lost time seeking diagnosis and care. Lost income was calculated using the human capital approach (Government of Nepal *et al.*, 2018), applying self-reported length of time absent from work, 2018 Nepali monthly minimum wage (US$121.05), the labour force participation rate (49%) and unemployment rate (1.2%)(World Bank, 2018). Lost time was converted to a monetary value by applying hourly (US$0.62) and daily (US$4.67) minimum wages (Government of Nepal *et al.*, 2018). Costs were collected in the local currency, NPR, and were converted to US$ applying the average exchange rate from OANDA during the data collection period (NPR 1 = US$0.009) (https://www1.oanda.com/) (OANDA, 2018). Participants who could not be located for the second or third interviews were considered lost to follow-up and were excluded from the analysis.

To evaluate uncertainty in costs, one-way sensitivity analysis was performed. Total costs were calculated by varying direct medical, non-medical and indirect costs according to the upper and lower limit of their CIs (Taylor, 2009).

#### Socio-economic impact

Income changes, employment status, poverty headcount (World Bank, 2019), self-reported social impact (food insecurity, social exclusion and others), self-reported sense of relative economic status (e.g. feeling poorer) and use of coping strategies were analysed throughout the treatment.

#### Catastrophic costs

The prevalence of households with catastrophic costs was determined for the WHO threshold for TB (total cost >20% of the annual household income). Catastrophic costs were calculated according to the annual household income self-reported before the onset of TB. Catastrophic costs and pretreatment costs were not calculated for retreatment and relapse TB cases as we were not able to accurately determine pretreatment costs for this group due to the length of time elapsed between initial TB diagnosis and the interview.

#### Statistical analysis

Data analysis was performed using Stata version 15 (STATA, Statacorp, TX, USA). Frequency distributions and descriptive statistics such as mean/median were calculated. Chained multiple imputation (Royston, 2005) was used to estimate missing costs data (Supplementary Table S1). Ten multiple imputed data sets with five iterations were generated. The variables gender, age, type of provider, district and ACF/PCF were included in the imputation model. Chi-square and Fischer’s exact test were applied to test differences in proportions of categorical variables. Wilcoxon rank-sum test was used to compare costs between ACF and PCF. *P*-values ≤0.05 were considered statistically significant.

The association between catastrophic costs and high costs, i.e. costs above the 75th quartile incurred during the pretreatment and treatment periods (de Cuevas *et al.*, 2016), and adjusted by baseline characteristics (i.e. ACF/PCF, sex, age, education level, employment status and patient income) was explored through multiple logistic regression. Odds ratios (ORs) with 95% CIs were estimated.

We fitted an interaction term between ACF/PCF and treatment phase using a generalized estimating equation (Zeger and Liang, 1986) to evaluate the effect of ACF on the socio-economic characteristics throughout TB treatment: unemployment, food insecurity, social exclusions, to be poorer/much poorer, coping strategies, patient and household incomes and poverty headcount.

We used the CHEERS (Husereau *et al.*, 2013) checklist when writing our report.

### Ethical

The study was approved by the ethical committees of the authors’ institutes. All participants received a written Patient Information Sheet and an oral explanation about the study. Written informed consent was obtained before each interview.

## Results

A total of 243 patients were recruited for the study. No eligible patient declined participation. Twenty-two patients (9%) were lost to follow-up, 20 patients were not located for the second interview and two for the third interview. Therefore, 221 patients completed the three interviews and were included in the final analysis: 111 ACF and 110 PCF. No deaths occurred among the included participants (Supplementary Figure S1). Included and excluded patients had similar socio-economic characteristics at baseline, except for income and ownership of bicycle (Supplementary Table S2).

### Socio-economic profile

Most participants were male (*n* = 147/221; 67%), in line with the gender ratio of notified TB cases in Nepal. ACF patients were more likely than PCF patients to be manual workers (28% vs 14%, *P* = 0.015), have a lower level of education, with significantly more individuals in the no-education category (75% vs 57%, *P* = 0.013) and significantly fewer having completed secondary school (9% vs 21%, *P* = 0.013). Ownership of a mobile phone and television was less frequent among ACF patients compared with PCF patients (88% vs 95%, *P* = 0.044 and 49% vs 63%, *P* = 0.042, respectively). Source of drinking water, type of toilet facility and availability of electricity in the home were similar among the ACF and PCF groups (Table 1).

**Table 1:** Baseline socio-economic characteristics of TB patients diagnosed through ACF and PCF. Nepal 2019

Patient features	ACF, *N* = 111	PCF, *N* = 110	Pooled sample, *N* = 221	*P*-value^a^
Sex, *N* (%)				
Male	71 (64)	76 (69)	147 (67)	0.42
Age, mean (SD)	50 (15)	46 (17)	48 (16)	0.057
Completed education, *N* (%)^b^				
No education	83 (75)	63 (57)	146 (66)	0.006*
Basic school	18 (16)	24 (22)	42 (19)	0.29
Secondary school	10 (9)	23 (21)	33 (15)	0.01*
Occupation, *N* (%)				
Farmer	23 (21)	16 (14)	39 (18)	0.23
Manual labour	31 (28)	16 (14)	47 (21)	0.01*
Unemployed	31 (28)	29 (26)	60 (27)	0.79
Others	26 (23)	49 (44)	75 (34)	0.001*
Patient income quartile				
Poorest	43 (39)	51 (46)	94 (43)	0.25
Moderately poor	13 (12)	6 (5)	19 (9)	0.10
Average	29 (26)	25 (23)	54 (24)	0.56
Wealthiest	26 (23)	28 (25)	54 (24)	0.72
Household income quartile				
Poorest	39 (35)	30 (27)	69 (31)	0.21
Moderately poor	21 (19)	23 (21)	44 (20)	0.71
Average	29 (26)	29 (26)	58 (26)	0.97
Wealthiest	22 (20)	28 (25)	50 (23)	0.32
Source of drinking water, *N* (%)				
Piped	34 (31)	40 (36)	74 (33)	0.37
Others	77 (69)	70 (64)	147 (67)	
Toilet facilities, *N* (%)^c^				
No toilets	25 (23)	16 (15)	41 (19)	0.13
Public sewage	1 (1)	5 (5)	6 (3)	0.10
Others	85 (77)	88 (81)	173 (79)	0.45
Electricity, *N* (%)	98 (91)	104 (94)	202 (93)	0.28
Assets, *N* (%)				
Mobile/phone	95 (88)	105 (95)	200 (92)	0.044*
Refrigerator	11 (10)	20 (18)	31 (14)	0.09
Television	53 (49)	69 (63)	122 (56)	0.042*
Radio	31 (29)	45 (41)	76 (35)	0.059
Bicycle	72 (67)	72 (65)	144 (66)	0.85
Motorbike	18 (17)	26 (24)	44 (20)	0.20
Livestock	80 (74)	76 (69)	156 (71)	0.41

a Chi-square and Fischer’s exact, Wilcoxon rank sum.

b Basic schools = primary level/lower secondary level (1–8 years of education).

c One missing data.

* Statistically significant

### Treatment characteristics

Most patients included in the study were new TB cases (214/221, 97%). During the pretreatment period, ACF patients reported less hospitalization (6% vs 19%, *P* = 0.004) and fewer visits to health providers (median number of visits = 2.8 vs 4.6, *P* < 0.001). ACF patients were less likely than PCF patients to visit public sector healthcare facilities (47% vs 55%, *P* = 0.026) and more likely to access other types of health providers in seeking a diagnosis, which includes local NGOs and informal providers such as pharmacists and traditional healers (25% vs 19%, *P* = 0.044). During the treatment period, the number of visits and type of health facilities visited were similar for both groups (Table 2).

**Table 2:** Treatment characteristics of TB patients diagnosed through ACF and PCF (Nepal, 2019)

Characteristics	ACF, *N* = 111	PCF, *N* = 110	Pooled sample, *N* = 221	*P*-value^a^
Treatment status, *N* (%)				
New	105 (95)	109 (99)	214 (97)	0.056
Retreatment and relapse	6 (5)	1 (1)	7 (3)	
HIV status, *N* (%)				
Positive	1 (1)	1 (1)	2 (1)	0.75
Negative	76 (68)	77 (70)	153 (69)	0.80
Unknown	34 (31)	32 (29)	66 (30)	0.99
Number of weeks between onset of TB symptoms and treatment initiation,^b^ median (IQR)	7 (3–13)	6 (4–12)	6 (3–13)	0.87
Hospitalization pretreatment,^b^*N* (%)				
Yes	7 (6)	21 (19)	28 (13)	0.004*
Hospitalization treatment, *N* (%)				
Yes	2 (2)	1 (1)	3 (1)	0.57
Visits to health providers, pretreatment^b^	ACF, *N* = 300^c^	PCF, *N* = 498^c^	Pooled sample, *N* = 798^c^	*P*-value^a^
Number of visits to health providers, mean (SD)	2.8 (1.8)	4.6 (2.3)	3.7 (2.2)	<0.001*
Type of service visited,^d^*N* (%)				
Public health centres/hospitals	140 (47)	273 (55)	413 (52)	0.026*
Private clinics/hospitals	84 (28)	129 (26)	213 (27)	0.52
Others^e^	76 (25)	96 (19)	172 (21)	0.044*
Visits to health providers, treatment^f^	ACF, *N* = 249^c^	PCF, *N* = 237^c^	Pooled sample, *N* = 486^c^	*P*-value^a^
Number of visits to health providers, mean (SD)	2.2 (1.2)	2.2 (1.3)	2.2 (1.3)	0.70
Type of service visited,^g^*N* (%)				
Public health centres/hospitals	208 (86)	203 (87)	411 (87)	0.70
Private clinics/hospitals	9 (4)	17 (7)	26 (5)	0.09
Others^d^	21 (10)	12 (5)	36 (8)	0.05

a Chi-square, Fischer’s exact and Wilcoxon rank sum.

b Six ACF and one PCF relapse cases excluded from the analysis.

c *N* is the total number of visits to health providers.

d One PCF visit missed.

e NGOs, and informal providers such as pharmacists and traditional healers.

f Emergency and inpatient care.

g Thirteen missing data.

### Patient costs

During the pretreatment period, ACF patients incurred lower total costs (mean cost, US$56 vs US$87, *P* < 0.001). When analysed by cost category, ACF patients also had significantly lower direct medical (mean cost, US$41 vs US$53, *P* < 0.001), non-medical (mean cost, US$7 vs US$18, *P* < 0.001) and indirect/time loss costs (mean cost, US$8 vs US$15, *P* < 0.001).

During the treatment period, the costs incurred by ACF and PCF patients were similar. The total costs incurred, including both pretreatment and treatment periods, was lower for ACF patients for direct medical (mean cost, US$58 vs US$74, *P* = 0.009), non-medical (mean cost, US$14 vs US$28, *P* < 0.001) and total direct cost (mean cost, US$72 vs US$101, *P* < 0.001) (Table 3).

**Table 3:** Mean and median costs per TB patient (US$) during pretreatment and treatment period in patients diagnosed through ACF and PCF (Nepal, 2019)

Cost item	ACF, *N* = 111		PCF, *N* = 110		Pooled sample, *N* = 221		*P*-value^a^
	Mean (95% CI)	Median (IQR)	Mean (95% CI)	Median (IQR)	Mean (95% CI)	Median (IQR)	
Pretreatment^b^							
Direct medical	41.1 (28.7–53.6)	12.3 (0–55.8)	53.1 (41.6–64.6)	29.6 (10.2–79.2)	47.2 (38.8–55.7)	21.7 (3.5–70.3)	<0.001*
Direct non-medical	6.8 (3.7–9.9)	1.4 (0–5.8)	18.4 (11.9–24.8)	5.3 (1.8–14.1)	12.7 (9.0–16.4)	3.0 (0.4–10.8)	<0.001*
Total direct pretreatment	47.9 (32.8–63.0)	13.3 (1.4–59.9)	71.5 (56.2–86.8)	40.9 (14.0–11.5)	59.9 (49.1–70.7)	28.4 (6.2–81.9)	<0.001*
Indirect, time loss	7.5 (5.6–9.5)	4.3 (1.9–8.7)	15.3 (11.9–18.6)	10.0 (5.6–18.0)	11.5 (9.4–13.5)	6.7 (3.3–13.6)	<0.001*
*Total pretreatment*	*55.5 (39.0*–*71.9)*	*20.4 (3.8*–*69.2)*	*86.7 (69.7*–*103.8)*	*58.2 (22.3*–*127.2)*	*71.4 (59.5*–*83.3)*	*33.6 (10.3*–*97.1)*	<0.001*
Treatment							
Direct medical	19.5 (13.5–25.5)	10.8 (6.3–20.7)	21.1 (14.9–27.4)	12.2 (7.2–21.2)	20.3 (16.0–24.6)	11.8 (6.6–20.7)	0.24
Direct non-medical	7.6 (5.1–10.2)	1.9 (0.3–9.4)	9.5 (6.8–12.1)	3.4 (0.7–13.2)	8.6 (6.7–10.4)	2.7 (0.7–10.8)	0.21
Total direct treatment	27.2 (20.2–34.1)	16.2 (9.5–31.1)	30.6 (23.3–37.9)	17.9 (12.0–37.7)	28.9 (23.9–33.9)	17.9 (10.7–32.3)	0.23
Indirect, time loss	38.7 (33.2–44.1)	29.6 (21.2–50.1)	44.1 (33.4–54.8)	27.9 (17.0–51.5)	41.4 (35.4–47.3)	29.1 (19.5–50.2)	0.54
*Total treatment*	*65.9 (55.1*–*76.6)*	*48.8 (35.4*–*78.5)*	*74.7 (60.6*–*88.8)*	*51.2 (29.9*–*91.7)*	*70.3 (61.5*–*79.1)*	*49.8 (33.2*–*83.4)*	*0.71*
Total costs (pretreatment + treatment)							
Direct medical	58.4 (45.4–71.4)	31.3 (11–73.3)	73.7 (60.0–87.5)	47.4 (21.9–102.9)	66.1 (56.6–75.5)	42.3 (18.0–87.9)	0.009*
Direct non-medical	14.1 (10.3–17.9)	6.5 (1.7–17.6)	27.7 (20.5–34.8)	15.2 (5.9–29.6)	20.8 (16.7–25.0)	10.6 (2.3–23.3)	<0.001*
*Total direct (A)*	*72.5 (57.2*–*87.8)*	*43.7 (21.70*–*92.5)*	*101.4 (83.6*–*119.2)*	*69.3 (39.2*–*136.9)*	*86.9 (75.1*–*98.7)*	*57.3 (27.4*–*112.9)*	<0.001*
Income loss	114.6 (90.0–139.2)	0 (0–250.6)	119.3 (88.4–150.2)	0 (0–263.7)	116.9 (97.4–136.5)	0 (0–251.4)	0.89
*Total indirect (B)*	*160.4 (135.8*–*185.1)*	*97.2 (42.5*–*286.5)*	*178.6 (146.4*–*210.7)*	*90.5 (46.7*–*302.4)*	*169.5 (149.3*–*189.6)*	*92.4 (42.9*–*296.2)*	*0.55*
*Total costs (A + B)*	*233.0 (204.6*–*261.4)*	*218.2 (97.1*–*340.6)*	*279.9 (244.8*–*315.2)*	*252.0 (117.9*–*393.3)*	*256.4 (233.7*–*278.9)*	*245.2 (113.1*–*365.6)*	*0.07*

a Wilcoxon rank sum.

b Six ACF and one PCF relapse and retreatment cases were not included in this analysis.

The multiple logistic regression showed that compared with PCF patients, ACF patients were 62% less likely to incur high total costs [adjusted OR = 0.38 (95% CI = 0.19–0.77)] (Supplementary Table S3).

The one-way sensitivity analysis showed that in both ACF and PCF, indirect costs were the parameter with highest uncertainty. For ACF patients, the total cost varied from US$208 to US$257 and for PCF the variation was from US$248 to US$312 (Supplementary Figure S2).

### Socio-economic impact and catastrophic costs

**Figure 1:**
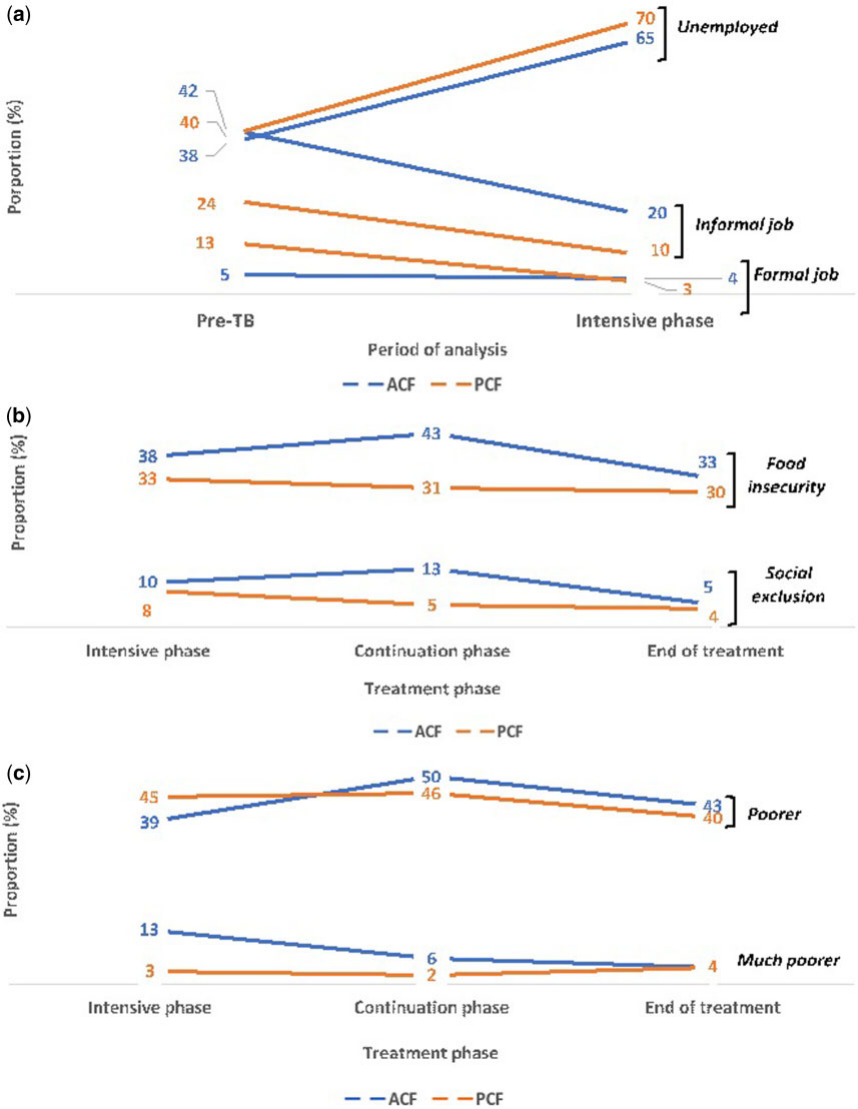
Socio-economic impact of TB in patients diagnosed through ACF and PCF according to the treatment phase (Nepal, 2019). (a) Employment status; (b) social impact; (c) financial impact; (d) coping strategies; (e) prevalence of catastrophic cost according to income quartile; (f) poverty headcount (%), median patient and household incomes (US$).

The proportion of patients unemployed increased compared with pretreatment employment status. This was true for both ACF and PCF patients (71% increase for ACF and 75% for PCF) (Table 4). ACF patients employed in formal jobs were less likely to change their employment status when compared with PCF (20% reduction in formal employment for ACF compared with 77% reduction for PCF) (Figure 1a).

**Table 4:** Socio-economic impact in patients diagnosed through ACF and PCF at different periods of analysis (Nepal, 2019)

Variables	Pre-treatment, *N* (%)		OR (95% CI)	Intensive phase, *N* (%)		OR (95% CI)	Continuation phase, *N* (%)		OR (95% CI)	End of treatment, *N* (%)		OR (95% CI)
	ACF, *N* = 111	PCF, *N* = 110		ACF, *N* = 111	PCF, *N* = 110		ACF, *N* = 111	PCF, *N* = 110		ACF, *N* = 111	PCF, *N* = 110	
Unemployed	42 (38)	44 (40)	0.91 (0.53–1.57)	72 (65)	77 (70)	0.79 (0.45–1.39)	ND	ND	ND	ND	ND	ND
Food insecurity	NA	NA	NA	42 (38)	36 (33)	1.25 (0.72–2.17)	48 (43)	34 (31)	1.70 (0.98–2.95) *	37 (33)	33 (30)	1.17 (0.66–2.06)
Social exclusion	NA	NA	NA	11 (10)	9 (8)	1.23 (0.49–3.11)	15 (13)	6 (5)	2.71 (1.01–7.26) *	6 (5)	4 (4)	1.51 (0.42–5.52)
Poorer/much poorer	NA	NA	NA	58 (52)	53 (48)	1.17 (0.69–1.99)	62 (54)	53 (46)	1.36 (0.80–2.31)	53 (52)	48 (48)	1.18 (0.69–2.00)
Coping strategies	NA	NA	NA	24 (22)	27 (25)	0.85 (0.45–1.59)	15 (13)	11 (10)	1.41 (0.61–3.21)	8 (7)	10 (9)	0.78 (0.29–2.05)
Patient income> median	55 (51)	53 (49)	1.06 (0.62–1.79)	32 (56)	25 (44)	1.38 (0.75–2.52)	37 (55)	30 (45)	1.33 (0.75–2.37)	38 (53)	33 (46)	1.21 (0.69–2.14)
Household income> median	51 (47)	57 (53)	0.79 (0.46–1.34)	48 (47)	55 (53)	0.76 (0.45–1.29)	51 (47)	58 (53)	0.76 (0.45–1.29)	51 (46)	59 (54)	0.73 (0.43–1.25)
Poverty headcount^a^	44 (40)	51 (46)	0.76 (0.44–1.29)	85 (77)	87 (79)	0.86 (0.45–1.63)	76 (68)	81 (74)	0.77 (0.43–1.39)	76 (68)	78 (71)	0.89 (0.50–1.81)

NA, not applicable; ND, no data.

a Poverty headcount: Proportion of patients living with less than $1.9 per day, International Dollar ($) calculated applying purchase power parity (PPP), 2018 prices, conversion factor = $34.93 (https://data.worldbank.org/indicator/PA.NUS.PPP? locations=NP).

Food insecurity was reported by over a third of households and was the social impact most frequently reported at all stages of TB treatment by both ACF patients (38%, 43% and 33%) and PCF patients (33%, 31% and 30%) (Figure 1b). ACF patients were more likely to report food insecurity [OR = 1.70 (95% CI = 0.98–2.95)] and social exclusion [OR = 2.71 (95% CI = 1.01–7.26)] during the continuation phase when compared with PCF patients (Table 4). Economically, over half of all patients reported feeling ‘poorer’ or ‘much poorer’, with no patients feeling ‘richer’ (Figure 1c).

The frequency and pattern of utilization of coping strategies was similar in the two groups in all treatment phases (Table 4). Approximately a quarter of patients reported using coping strategies, i.e. selling essential assets or taking out loans, during the intensive phase of treatment (22% for ACF and 25% for PCF, *P* = 0.61) and the frequency had reduced by the continuation phase (13% for ACF and 10% for PCF, *P* = 0.42) and treatment completion (7% for ACF and 9% for PCF, *P* = 0.61) (Figure 1d). The prevalence of catastrophic costs was similar for ACF and PCF (31% vs 32%, *P* = 0.91) and more frequent in the poorest households (Figure 1e). ‘No education’ was associated with catastrophic costs [adjusted OR = 2.84 (95% CI = 1.34–6.00)] (Supplementary Table S4).

Patient income, household income and poverty headcount trends were similar for ACF and PCF throughout treatment (Table 4). The median patient income decreased to US$0 during the intensive phase for both groups and patients did not recover their pre-TB income by the end of treatment. The same pattern was observed for household income. In the intensive phase, the poverty headcount increased from 40% to 77% for ACF (92% increase) and from 46% to 79% for PCF (72% increase). The poverty headcount remained high until the end of treatment for both groups (Figure 1f).

## Discussion

The economic consequences of TB disease for affected families can be devastating. Our data showed that the mean total costs incurred were US$256 in a country with a Gross National Income per capita of US$970 in 2018 (World Bank, 2018). Three quarters of TB patients experienced extreme poverty in the intensive phase of treatment. Importantly, we have shown that ACF can be an effective strategy to both reach the most vulnerable patient groups and reduce the economic impact.

ACF patients diagnosed under the community-based strategy were more likely to be those with no formal education, working in the informal sector and in the lowest socio-economic groups. These are the patients failed by the standard model of NTPs using PCF (Li *et al.*, 2013; Ukwaja *et al.*, 2013).

These findings add to the body of evidence showing that ACF strategies can increase equity of access to TB services, particularly among the most vulnerable and disadvantaged populations, and bring us closer to achieving the Declaration of Rights for TB Patients (Yassin *et al.*, 2013; The Global Fund, 2018; Saunders *et al.*, 2019). Studies conducted in India and Nigeria also found higher vulnerability among ACF patients when compared with PCF, such as lower education level, higher rates of unemployment, older patients and longer duration of symptoms (Abdurrahman *et al.*, 2017; Shewade *et al.*, 2018).

We also demonstrated that ACF was associated with significantly lower patient costs during the pretreatment period (mean total pretreatment costs US$56 for ACF group vs US$87 for PCF group; *P* < 0.001). Cost surveys conducted in Nepal and Cambodia found similar results as ours (Morishita *et al.*, 2016; Gurung *et al.*, 2019). Although the number of weeks between the first TB symptoms and treatment initiation was similar between ACF and PCF, ACF patient costs were mitigated by the reduction in the number of visits to health facilities during the pretreatment period and, consequently, reduction in direct cost, such as transportation, unnecessary medication and tests in private services, and time lost waiting for appointments and traveling to and from healthcare facilities. ACF would not be expected to substantially influence the patient costs once on treatment, since both patient groups were enrolled into and treated via the government DOTS programme.

ACF was associated with lower total costs [adjusted OR = 0.38 (95% CI = 0.19–0.77)]. However, the prevalence of catastrophic costs was similar for both ACF and PCF patients, reflecting the lower initial socio-economic status of the ACF group. Also, our data showed that catastrophic costs were associated with ‘no education’ status, which was more frequent in ACF patients. Other studies have found catastrophic costs associated with number of symptoms, number of healthcare visits and use of nutritional supplements in South Africa (Foster *et al.*, 2015); alcohol use and PCF in India (Muniyandi *et al.*, 2020); and previous TB treatment and job loss in Indonesia (Fuady *et al.*, 2018). Our findings strengthen the evidence that while ACF may reduce household expenditure, this strategy needs to be implemented alongside social protection policies to protect TB patients from financial hardship and to achieve the zero catastrophic costs target in by the End TB strategy (World Health Organization, 2015b).

The longitudinal design identified a similarly severe pattern of socio-economic impact throughout the treatment for both ACF and PCF groups. The disease caused major socio-economic consequences for patients during the intensive phase. These included increased unemployment, a drastic reduction in income, high rates of food insecurity, utilization of coping strategies and falling into extreme poverty. Patients continued to report high rates of financial and social impact at treatment completion and were not able to recover the income to the levels earned before the onset of TB symptoms. These findings indicate that TB triggered the medical poverty trap mechanism, which reinforces the poverty cycle and can persist for generations (Whitehead and Dahlgren, 2007; Barrett and Carter, 2013; Wingfield *et al.*, 2014). Further studies adopting a longer follow-up are needed to evaluate the socio-economic impact post-TB treatment.

One of the limitations of our study is recall bias which may have affected the accurate estimation of costs and catastrophic cost due to the long interval between the interviews. The literature has shown that recall bias particularly affects the estimates of indirect costs, income lost in developing countries (World Health Organization, 2017; Stracker *et al.*, 2019). The same is true in Nepal, where the majority of TB patients are employed in the informal market or in seasonal jobs that do not provide regular salaries or payslips (World Bank, 2018). Also, the prevalence of catastrophic costs by using self-reported income can be underestimated when compared with methods such as the asset linking approach or income estimated using the national average (Sweeney *et al.*, 2018).

The IMPACT TB costing survey was the first longitudinal survey comparing ACF and PCF strategies in Nepal. The study design allowed the investigation of the socio-economic impact of TB throughout the whole treatment. Another advantage of this approach is the continuous collection of costing data with no extrapolation techniques (WHO, 2017) applied, which will increase the accuracy of estimates compared with the cross-sectional methodology. Another limitation of this study was the missing pretreatment costs for relapse and retreatment patients with possible underestimation of the total cost for ACF as this intervention had more patients in this treatment category (6 ACF vs 1 PCF). However, a cross-sectional survey conducted in Nepal indicated that PCF patients were more likely to be affected by memory bias and underestimate costs for pretreatment and intensive phase. A sensitivity analysis comparing costs reported by ACF and PCF patients interviewed within and after 1 month of treatment initiation found that PCF patients reported lower median total costs when interviewed after 1 month after starting treatment during the pretreatment period(<1 month: US$ 365.9; >1 month: US$ 128.5; *P* = 0.007), intensive phase of treatment (<1 month: US$ 190.4; >1 month: US$ 67.6; *P* = 0.004) and total costs estimates (<1 month: US$ 556.3; >1 month: US$ 232.3, *P* = 0.002). No difference in costs was reported for ACF patients interviewed within or after 1 month of treatment initiation (Gurung *et al.*, 2019). Therefore, the missing cost in the pretreatment period for relapse and retreatment patients is unlikely to have affected the differences in total costs between ACF and PCF found in our survey.

ACF has been sporadically implemented in Nepal through several organizations and using different approaches (Stop TB Partnership and Birat Nepal Medical Trust, 2015; The Global Fund, 2018). However, to achieve a comprehensive and sustained implementation of efficient ACF models, some priority actions must be in place. These actions must consider the limited health system resources and the complex geographical features of Nepal. Improvements to human resource training and retention, an efficient quality control and logistics system to support diagnostic centres and reduction of import duties on advanced molecular TB diagnostic tests such as GeneXpert (Siqueira *et al.*, 2019) would facilitate scale-up of ACF. The use of innovative technologies, such as drones to collect sputum sample and deliver TB medications, will be crucial to address challenges in sample transportation and comprehensively reach vulnerable communities in hard to reach areas (Pudasaini, 2019; Birat Nepal Medical Trust, 2020). Improved public–private linkages will also be essential to improve patient access to high-quality TB diagnosis and treatment and to bring ACF to the healthcare facility level (Government of Nepal *et al.*, 2016). From the NTP perspective, an efficient allocation of human and financial resources and improvement of existing diagnostic centres are essential to successfully scale-up ACF in Nepal (Ministry of Health & Population *et al.*, 2018).

To break the poverty cycle among TB patients, alleviation programmes such as cash transfer, nutritional support and livelihood rehabilitation schemes must be accessible to patients from diagnosis and work in synergy with government scale-up initiatives under the SDG drive for Universal Health Coverage.

## Conclusions

The community-based ACF model reached the most vulnerable patients and significantly reduced patient costs in the pretreatment phase. However, patients in both the ACF and PCF groups reported severe and enduring socio-economic consequences. Therefore, policies including social protection must be implemented to reach the End TB strategy goals.

## Supplementary data

Supplementary data are available at *Health Policy and Planning* online.

## Funding

The study was supported by European Union Horizon2020 [grant number 733174: IMPACT TB].

## Supplementary Material

czaa156_Supp
