# Supplementary material for: How to reduce household costs for people with tuberculosis: a longitudinal costing survey in Nepal
Source: Health Policy Plan. 2020 Dec 20;36(5):594–605. doi: 10.1093/heapol/czaa156 (PMC8173598; doi:10.1093/heapol/czaa156)
Supplement: czaa156_Supp [file czaa156_supp.zip › czaa156_Supplementary_Data.docx]

**Supplementary material**

Figure S1. Study flow chart. Nepal, 2019.

Table S1. Missing values imputed using the chained imputation approach

| **Variable** | **Number of imputations** | | | |
| --- | --- | --- | --- | --- |
|  | **Pre-treatment** | | **Treatment** | |
|  | **ACF**  **N= 300^1^** | **PCF**  **N= 498^1^** | **ACF**  **N= 249^1^** | **PCF**  **N= 237^1^** |
| Days charge | 8 | 10 | 0 | 0 |
| Consultation | 15 | 30 | 5 | 0 |
| Radiography | 20 | 31 | 5 | 1 |
| Laboratory tests | 19 | 45 | 4 | 2 |
| Other procedures | 4 | 17 | 0 | 0 |
| Medicines | 24 | 41 | 3 | 0 |
| Other direct medical | 32 | 65 | 6 | 1 |
| Transportation | 7 | 1 | 3 | 0 |
| Food | 9 | 7 | 3 | 0 |
| Other direct non-medical | 7 | 5 | 3 | 0 |

^1^ Number of visits to health services.

Table S2. Baseline socio-economic characteristics of TB patients included and excluded from the study. Nepal 2019.

| **Patient Features** | **Patients included**  **N=221** | **Patients excluded**  **N=22** | **P-value¹** |
| --- | --- | --- | --- |
| **Sex, N (%)** |  |  |  |
| Male | 147 (67) | 12 (55) | 0.260 |
| **Age, mean (SD)** | 48 (16) | 44 (16) | 0.2389 |
| **Completed education, N (%)^2^** |  |  |  |
| No education/Basic school | 188 (85) | 21 (95) | 0.180 |
| Secondary school | 33 (15) | 1 (4) |  |
| **Occupation** |  |  |  |
| Farmer | 39 (18) | 4 (18) | 0.950 |
| Manual labour | 15 (7) | 1 (5) | 0.686 |
| Unemployed | 80 (36) | 5 (23) | 0.206 |
| Others | 87 (39) | 12 (55) | 0.167 |
| **Patient income, median (IQR)** | 45 (0-135) | 110 (45-162) | 0.029* |
| **Household income, median (IQR)** | 153 (90-270) | 184 (90-243) | 0.776 |
| **Source of drinking water, N (%)** |  |  |  |
| Piped | 74 (33) | 8 (36) | 0.785 |
| Others | 147 (67) | 14 (64) |  |
| **Toilet facilities, N (%)^3^** |  |  |  |
| No toilets | 41 (19) | 5 (24) | 0.564 |
| Public sewage | 6 (3) | 0 | 0.443 |
| Others | 173 (79) | 16 (76) | 0.795 |
| **Electricity, N (%)** | 202 (91) | 19 (86) | 0.432 |
| **Assets, N (%)** |  |  |  |
| Mobile phone | 200 (92) | 18 (82) | 0.124 |
| Refrigerator | 31 (14) | 2 (9) | 0.506 |
| Television | 122 (56) | 10 (45) | 0.345 |
| Radio | 76 (35) | 7 (32) | 0.775 |
| Bicycle | 144 (66) | 8 (36) | 0.006* |
| Motorbike | 44 (20) | 3 (13) | 0.461 |
| Livestock | 156 (71) | 12 (57) | 0.167 |

¹ Chi square, Fischer exact, Wilcoxon rank sum; ² Basic schools= primary level/lower secondary level (1 to 8 years of education); ^3^ One missing data.

Table S3. Association between high median costs (total costs higher than the 75^th^ quartile) and baseline characteristics.

| **Baseline characteristics** | **Crude**  **OR (95%CI)** | **Adjusted**  **OR (95%CI)** |
| --- | --- | --- |
| Variables | |  |
| ACF | 0.45 (0.24-0.85)* | 0.38 (0.19-0.77)* |
| Female | 0.51 (0.26-1.03) | 0.99 (0.42-2.35) |
| Age^1^ | 1.64 (0.88-3.06) | 1.96 (0.97-3.95) |
| Patient income^2^ | 3.57 (1.85-6.88)* | 4.74 (2.09-10.76)* |
| No education | 1.11 (0.58-2.12) | 1.58 (0.73-3.41) |
| Manual labour | 1.52 (0.75-3.08) | 0.99 (0.42-2.31) |

^1^ Age>48 years (mean age for pooled sample)

^2^ Income >US$45 (median pre-TB income for pooled sample)

Table S4. Association between catastrophic costs and baseline characteristics.

| **Baseline characteristics** | **Crude**  **OR (95%CI)** | **Adjusted**  **OR (95%CI)** |
| --- | --- | --- |
| Variables | |  |
| ACF | 0.97 (0.54-1.72) | 0.77 (0.42-1.44) |
| Female | 0.78 (0.42-1.45) | 0.72 (0.36-1.44) |
| Age^1^ | 2.10 (1.16-3.80)* | 1.75 (0.92-3.32) |
| No education | 2.87 (1.44-5.71)* | 2.84 (1.34-6.0)* |
| Manual labour | 1.53 (0.77-3.08) | 1.65 (0.73-3.69) |

^1^ Age>48 years (mean age for pooled sample)

Figure S2. One-way sensitivity analysis varying total direct medical, non-medical and indirect, costs. Nepal, 2019.

a) Active case finding

b) Passive case finding
